# Altered Cell Mechanics from the Inside: Dispersed Single Wall Carbon Nanotubes Integrate with and Restructure Actin 

**DOI:** 10.3390/jfb3020398

**Published:** 2012-05-23

**Authors:** Brian D. Holt, Hengameh Shams, Travis A. Horst, Saurav Basu, Andrew D. Rape, Yu-Li Wang, Gustavo K. Rohde, Mohammad R. K. Mofrad, Mohammad F. Islam, Kris Noel Dahl

**Affiliations:** 1 Department of Biomedical Engineering, Carnegie Mellon University, Pittsburgh, PA 15213, USA; Email: bholt@andrew.cmu.edu (B.D.H.); thorst@andrew.cmu.edu (T.A.H.); sauravb@cmu.edu (S.B.); arape@andrew.cmu.edu (A.D.R.); yuliwang@andrew.cmu.edu (Y.L.W.); gustavor@cmu.edu (G.K.R.); 2 Department of Bioengineering, University of California, Berkeley, CA 94720, USA; Email: hengameh@Berkeley.edu (H.S.); mofrad@berkeley.edu (M.R.K.M.); 3 Department of Chemical Engineering, Carnegie Mellon University, Pittsburgh, PA 15213, USA; 4 Center for Bioimage Informatics, Carnegie Mellon University, Pittsburgh, PA 15213, USA; 5 Department of Materials Science and Engineering, Carnegie Mellon University, Pittsburgh, PA 15213, USA

**Keywords:** actin, cytoskeleton, carbon nanotube, myosin, FLIM, cell mechanics

## Abstract

With a range of desirable mechanical and optical properties, single wall carbon nanotubes (SWCNTs) are a promising material for nanobiotechnologies. SWCNTs also have potential as biomaterials for modulation of cellular structures. Previously, we showed that highly purified, dispersed SWCNTs grossly alter F-actin inside cells. F-actin plays critical roles in the maintenance of cell structure, force transduction, transport and cytokinesis. Thus, quantification of SWCNT-actin interactions ranging from molecular, sub-cellular and cellular levels with both structure and function is critical for developing SWCNT-based biotechnologies. Further, this interaction can be exploited, using SWCNTs as a unique actin-altering material. Here, we utilized molecular dynamics simulations to explore the interactions of SWCNTs with actin filaments. Fluorescence lifetime imaging microscopy confirmed that SWCNTs were located within ~5 nm of F-actin in cells but did not interact with G-actin. SWCNTs did not alter myosin II sub-cellular localization, and SWCNT treatment in cells led to significantly shorter actin filaments. Functionally, cells with internalized SWCNTs had greatly reduced cell traction force. Combined, these results demonstrate direct, specific SWCNT alteration of F-actin structures which can be exploited for SWCNT-based biotechnologies and utilized as a new method to probe fundamental actin-related cellular processes and biophysics.

## 1. Introduction

Single wall carbon nanotubes (SWCNTs) are composed of sp^2^ bonded carbon atoms that are arranged into a quasi one-dimensional cylindrical geometry, with typical diameters ranging from 0.7–2 nm and typical lengths ranging from 20 nm–20 cm [1,2]. SWCNTs’ unique structure leads to numerous desirable properties, including high mechanical strength [3], surface area-to-volume ratio [2] and electrical conductivity [4,5], low mass density [2], resistance to chemical degradation [1,2] and unique optical properties [2,6]. These properties are suited for biomedical applications, and SWCNTs show promise as intracellular biomaterials since they are unreactive and resistant to acids, bases, enzymes, mechanical fatigue and temperature. Currently, SWCNT-based biomedical applications include use as biosensors [7], interfaces for microelectronic devices [8], tissue imaging agents [9,10,11], actuation materials [12], delivery vehicles for nucleic acids [13,14,15,16] and drugs [10,14,17,18,19,20,21,22], photothermal therapy agents [23], materials to pattern tissue culture substrates and control cell growth [24,25] and enhancers for tissue engineering matrices [26]. We suggest that SWCNT interactions with intracellular components may allow for the advancement of cellular medical technologies.

Bare SWCNTs are hydrophobic and readily flocculate due to van der Waals forces [27], and a “dispersing agent” is needed to stabilize individual SWCNTs in aqueous solutions. There are numerous dispersing agents capable of generating biocompatible SWCNTs for delivery applications. We have shown that SWCNTs dispersed with a biocompatible and FDA-approved triblock co-polymer, Pluronic F127 (PF127) [28], (SWCNTs-PF127) enter cells via endocytosis [29] and cause changes to filamentous actin (F-actin) structures inside the cell [30]. F-actin plays an important part in cellular motility, division, force generation and structure, as well as having many other indirect roles within the cell [31]. Modulation of the actin cytoskeleton and intracellular force generation within the cell also alters cell phenotype and cell differentiation [32,33,34,35,36,37].

SWCNTs represent a new, synthetic actin modifier, which causes *in situ* bundling and redistribution of F-actin. We have previously shown that high levels of SWCNT-PF127 treatment are associated with giant cells, multi-nucleated cells and cells frozen in cytokinesis [30]; therefore, high concentrations of SWCNTs could be used to generate populations of cells with F-actin-related defects. In this work, we investigated SWCNTs/actin interaction at the molecular, sub-cellular, cellular and functional levels. We demonstrated via molecular dynamics simulation that SWCNTs bind F-actin, and we showed the molecular details and strengths of binding partnership between the two. Furthermore, we investigated the interactions between actin monomers in association with SWCNTs. We probed SWCNT-actin interactions inside cells using fluorescence lifetime imaging microscopy (FLIM), further suggesting that SWCNTs are preferentially associated with F-actin structures. The altered organization of intra-cellular F-actin structures showed a reduction in filament length and an increase in apical protrusions. However, there was no effect on myosin II in the presence of SWCNTs-PF127. Functionally, incubation of fibroblasts with SWCNTs-PF127 resulted in an isotropic reduction in cellular generation of traction force. Therefore, we confirmed direct SWCNT interaction with F-actin, and we suggest the utility of SWCNTs as a new actin modifier and potential actuator.

## 2. Results and Discussion

### 2.1. SWCNTs Alter F-Actin Structure

We have shown that SWCNTs-PF127 enter cells via endocytosis, and we suggest that the membrane active PF127 disrupts the endosome, allowing SWCNT association with subcellular structures [29,30]. Many independent groups have shown that F-actin and focal adhesion structures are altered in cells treated with carbon nanotubes [38,39,40,41,42,43,44]. However, systematic investigations of SWCNTs with actin using complementary *in vitro* and *in situ* methods have allowed us to examine actin reorganization in cells and subsequent altered cellular phenotype. 

#### 2.1.1. Actin Filament Redistribution in Human Cells

Previously, we have visualized F-actin structures in SWCNTs-PF127-treated HeLa cells using confocal fluorescence microscopy [30]. In individual cells, actin filaments labeled with rhodamine phalloidin were mislocalized throughout the cell: (1) actin filaments throughout the height of the cell; (2) short peri-nuclear actin filaments; (3) “fuzzy” actin filaments at the apical surface of the cell, which are likely similar to; (4) actin protrusions extending radially from the edge of the cells. In addition to these localized changes in F-actin, we also observed global reordering of F-actin structure and a loss of cellular anisotropy. These changes, and the actin changes described below, were associated with exceptionally high levels of SWCNT treatment (>30 μg/mL); these concentrations are 1–2 orders of magnitude higher than treatment levels for SWCNT-based delivery systems.

**Figure 1:**
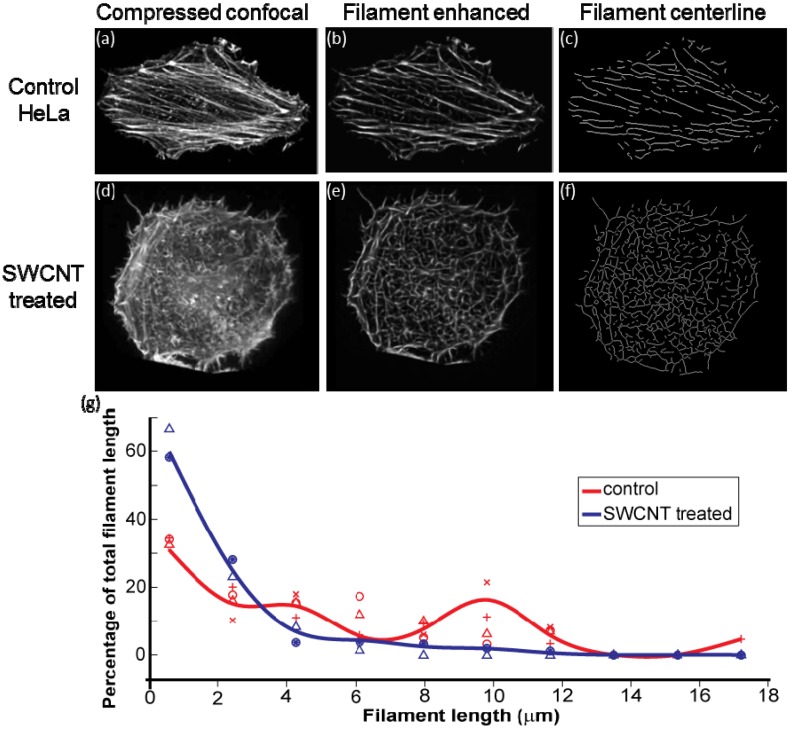
Morphometric image analysis of F-actin treated with 100 μg/mL SWCNTs-PF127: (**a**) Rhodamine phalloidin stained F-actin in control cell; (**b**) Filament enhanced image from (a) emphasizing filaments and suppressing background fluorescence; (**c**) Centerline of filaments obtained from (b); (**d**), (**e**) and (**f**) Corresponding images of (a), (b) and (c) with the entire process applied to a cell treated with SWCNTs-PF127; (**g**) Frequency distribution of actin filament length as determined from the filament centerline images (n = 4 control cells, n = 2 SWCNT-treated cells). Weighted average lengths are 4.40 +/− 0.49 μm for control and 1.66 +/− 0.41 μm for SWCNT-treated (*p* < 0.01).

Here, we have applied a sophisticated image analysis algorithm to quantify F-actin differences within the cell from standard 2-dimensional images. This study examines completely isolated HeLa cells treated with extremely high levels of SWCNTs (100 μg/mL SWCNTs-PF127 for 24 hr) to push the limit of cell response and remove confounding effects from cell-cell interactions. From compressed confocal images of rhodamine-phalloidin-labeled F-actin of HeLa cells (Figure 1a,d), we first compared the raw images against a bank of artificial linear and curvilinear elements. This step enhanced the filaments and suppressed the background noise and fluorescence contributed by un-polymerized actin, subsequently generating a filament enhanced image (Figure 1b,e). From there, we applied binary clustering followed by a morphological thinning step on the filament enhanced images to determine the centerlines of actin structures (Figure 1c,f). After disambiguating centerline bifurcations and intersections and measuring the centerline lengths of the resultant network, we observed a statistically significant reduction in filament length from many samples: 4.40 +/− 0.49 μm for control to 1.66 +/− 0.41 μm for SWCNT treated (*p* < 0.01, Figure 1g). Also, the multimodal length features of typical actin filaments are lost in the presence of SWCNTs.

**Figure 2:**
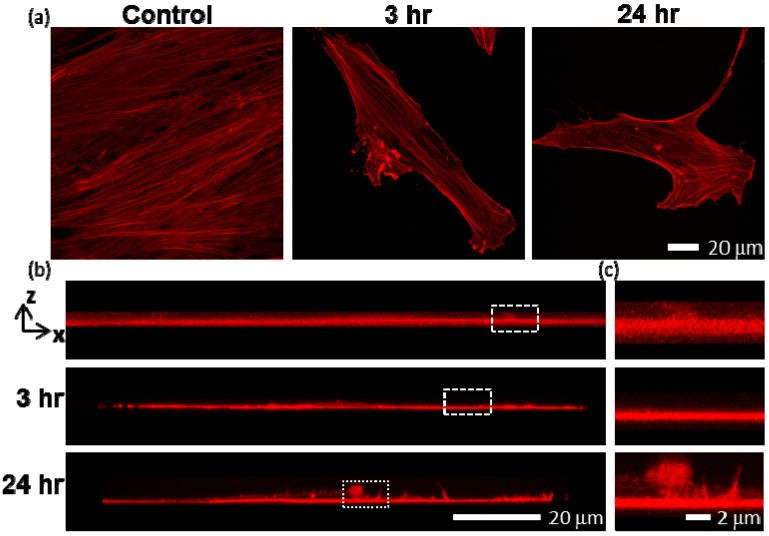
Confocal images of rhodamine phalloidin-labeled F-actin of hMSCs exposed to SWCNTs at 30 μg/mL; (**a**) Confocal compressions in Z show regions of altered F-actin structures after only 3 hr of SWCNT exposure; (**b**) Confocal compressions in Y reveal that after 3 hr of exposure, the areas of F-actin mislocalization did not substantially extend in the Z-direction; however, after 24 hr of exposure, the projections in Z were overt and substantial; (**c**) Zoomed in view of (b).

Recently, we reported that human mesenchymal stem cells (hMSCs) exhibited a 5–10× increased SWCNT uptake compared with other cell types, including NIH-3T3 cells and HeLa cells, from the initial study [45], possibly due to increased metabolic activity [46,47]. Treatment of hMSCs with 30 μg/mL SWCNTs-PF127 also showed redistribution of F-actin (Figure 2). SWCNTs-PF127-treated hMSCs were associated with misaligned actin filaments after only 3 hr of high levels of exposure (Figure 2a). After 24 hr, regions of misaligned, isotropic F-actin were extended vertically in dramatic fashion (Figure 2b, c (zoomed)). 

**Figure 3:**
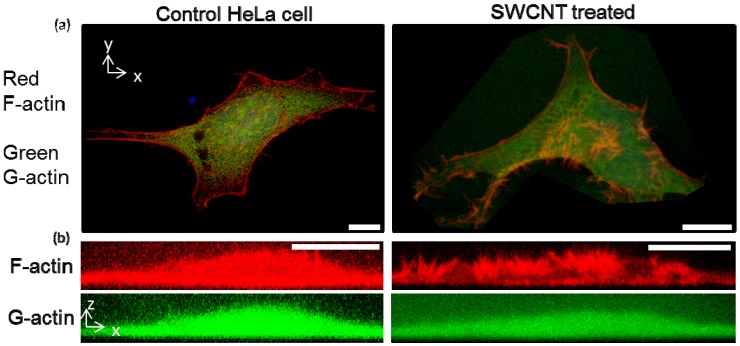
Confocal images of rhodamine phalloidin-labeled F-actin, Alexa Fluor 488 DNase I-labeled G-actin and DAPI-labeled nuclei (weak blue color) of control and SWCNTs-PF127-treated HeLa cells: (**a**) Confocal compressions in Z; (**b**) Confocal compressions in Y. Scale bars are 10 μm.

HeLa cells with neighboring cells treated with 30 μg/mL SWCNTs-PF127 showed cellular redistribution of F-actin that was less dramatic than high levels of treatment on isolated cells (Figure 3 compared with Figure 1). Generally, we have found that isolated cells (or cells at lower seeding density) were more sensitive to SWCNT treatment and showed a more dramatic response [30]. Here, confocal imaging of F-actin structures inside cells showed redistribution from the basal region of the cell to spiky apical plaques, changing cell distribution, global structures and localized structures. From the X-Y view (Figure 3a), small F-actin projections are observed at the periphery of the cell. However, there was no alteration to G-actin, suggesting a specific interaction between SWCNTs and F-actin.

#### 2.1.2. FLIM Shows F-actin-SWCNT Interactions *in Vitro* and *in Situ*

**Figure 4:**
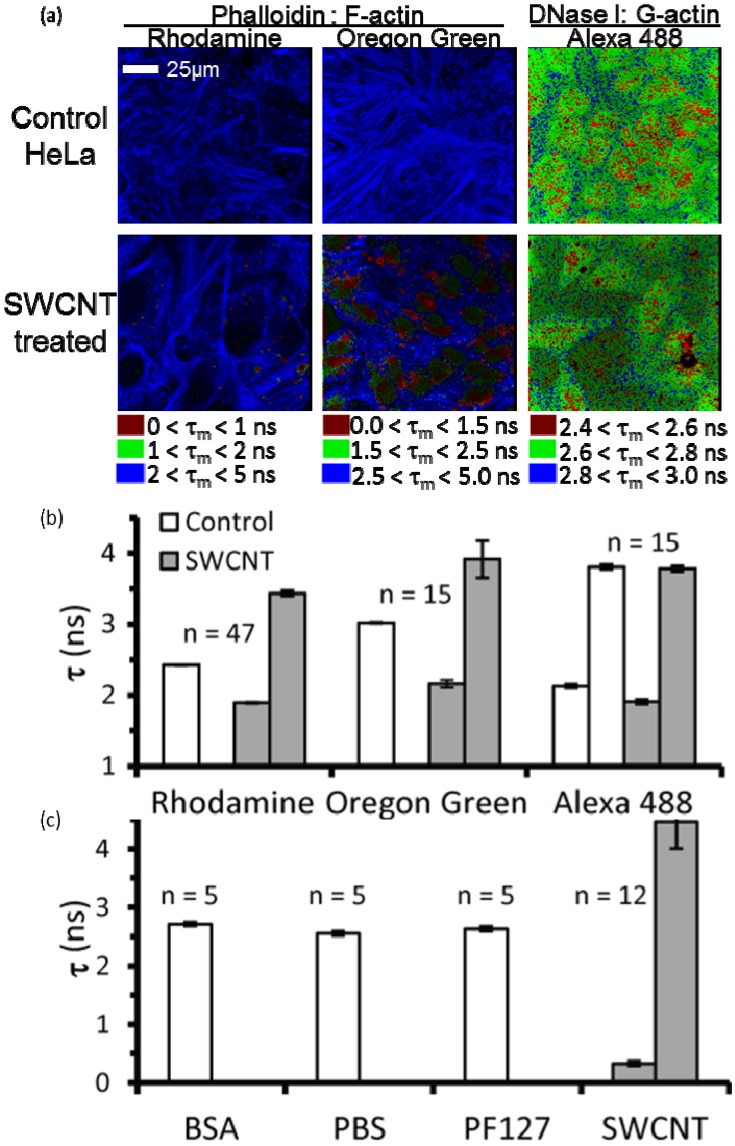
FLIM lifetime images and quantification of averaged mean fluorescence lifetimes (τ_m_); (**a**) τ_m_ pseudo-color images of HeLa cells labeled with rhodamine phalloidin (left), Oregon Green phalloidin (middle) and Alexa Fluor 488 DNase I. (Note: τ_m_ = τ_1_ for a single exponential decay); (**b**) Quantification of (a), showing the individual lifetime time constants, τ_1_ and τ_2_. The exponential model (single or double) was determined by the model having the goodness of fit (χ^2^) closest to unity; (**c**) Quantification of fluorescence lifetime images from *in vitro* samples, as in (b). Note: the value of n reflects the number of images averaged, each of which consists of 256×256 pixels, each of which has a value of fluorescence lifetime (if signals were present).

To examine more direct interactions of SWCNTs-PF127 with actin, we used fluorescence lifetime imaging microscopy (FLIM) to visualize potential fluorescence lifetime quenching in the presence of SWCNTs (Figure 4). FLIM measures changes in the fluorescence emission decay time and is highly sensitive to environmental factors within the Föster radius (~5 nm) of the fluorophore, including electronic interactions with SWCNTs [48]. Fluorescence lifetime, dependent on fluorophore and chemistry within the Föster radius, can be modeled as a single or double exponential decay [48].

We performed FLIM of rhodamine phalloidin labeled F-actin structures inside SWCNTs-PF127-treated HeLa cells. SWCNTs quenched the mean fluorescence lifetime (τ_m_) of the rhodamine, suggesting a close interaction (Figure 4a,b). To ensure that the quenching effects were due to SWCNT interaction with F-actin *versus* a SWCNT interaction with the fluorophore, we performed FLIM with Oregon Green phalloidin labeled actin filaments (Figure 4a,b). In both cases, quenching of τ_m_ was localized within specific F-actin regions (Figure 4a). Also, both cases showed a quantitative change in fluorescence lifetime profile (based on a goodness of fit, χ^2^, closest to unity), shifting from a single exponential decay for control to a double exponential decay for SWCNT-treated samples (Figure 4b). Interestingly, we observed no change in τ_m_ for Alexa Fluor 488 DNAse I (Figure 4a,b), which labels the monomeric G-actin in cells. Spatially, some fluctuations were observed in a very small range (~500 ps), but on average there was no change in the τ_m_ (Figure 4b). 

These studies suggest that SWCNTs show specific association with F-actin inside cells. To confirm SWCNT-actin filament specificity, we polymerized purified actin *in vitro* and treated purified actin filaments with bovine serum albumin (BSA), higher salt (with phosphate buffered saline, PBS), the SWCNT-dispersion polymer PF127 and PF127-dispersed SWCNTs (Figure 4c). The fluorescence lifetime of rhodamine phalloidin-labeled F-actin was only altered for SWCNT-PF127 (Figure 4c). Collectively, these results suggest a preferential interaction of SWCNTs with F-actin, both inside the cell and *in vitro*. 

#### 2.1.3. Molecular Interactions with Filamentous Actin

**Figure 5:**
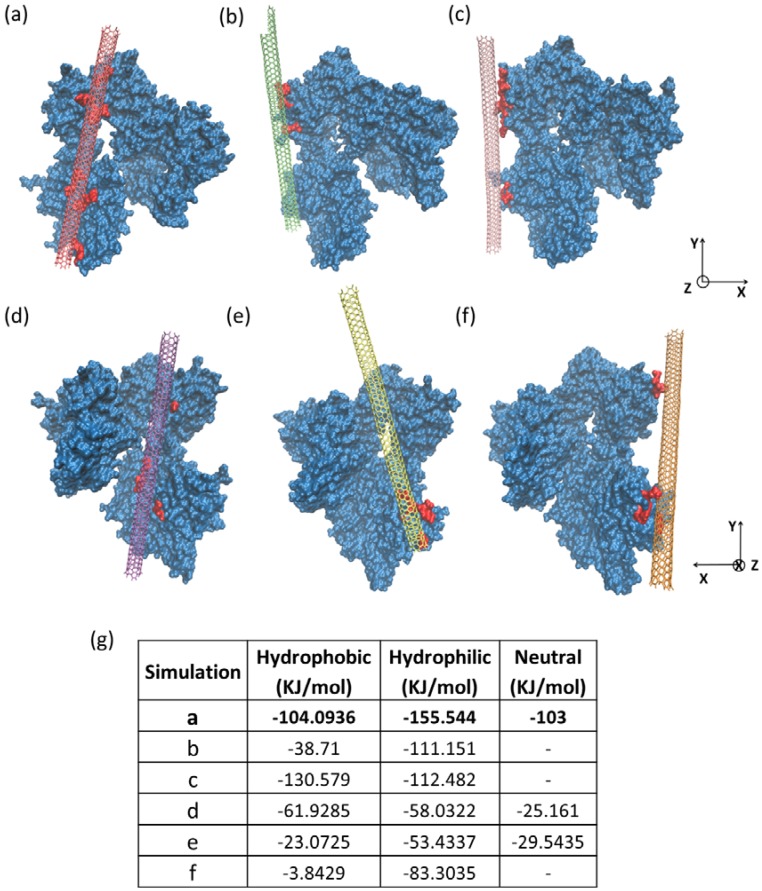
Molecular dynamics simulations of SWCNT interactions with a three actin monomer representation of F-actin: (**a**–**f**) represent different starting orientations between the actin and SWCNT, leading to different binding positions, the energies of which are indicated in (**g**); Red represents the interaction sites in all figures; (**a**) corresponds to the simulation with the lowest interaction energy, *i.e.*, the largest number of interactions while (**f**) has the lowest potential energy since the strong association of actin monomers was less disturbed compared to other simulations.

Although there is no unique actin-SWCNT binding site, as there is for specific protein-protein binding interfaces, we examined the interfacial energy to determine pseudostable binding regions. We simulated model F-actin filaments interacting with SWCNTs to determine possible mechanisms of interactions. We also considered specific interactions of F-actin using molecular dynamics simulations of three actin monomers combined in a filamentous arrangement (Supplementary Information, Figure 1) as a model segment of F-actin (Figure 5). To explore potential binding areas on the exposed surface of F-actin, we designed and performed six simulations, each having one SWCNT aligned parallel to the actin subunit (two actin monomers) but at different locations with respect to others (Figure 5a–f).

Initial configuration of all simulations started from SWCNT-actin distances within 3–5 angstroms, and we observed that the potential energy of the bound structure was optimized with a stable contact surface area. During all simulations, SWCNTs slid constantly on the surface of actin structures along the length of the filament, showing that the dynamic nature of SWCNT was maintained even in the bound state (for further details, please see Shams *et al*. [49]).

In all six simulations, the surface residues of actin formed strong interactions with the SWCNT. There were no electrostatic interactions between protein residues and SWCNTs since graphene bonds are neutral. Therefore, hydrophobic and π-π interactions were most significant in stabilizing the SWCNT on the surface of F-actin. Although the hydrophobic residues were mainly buried inside the core of actin monomers, there were a few on the surface, which were important for SWCNT binding such as ILE4, VAL95, PRO111, PRO331 *etc*. Aromatic residues, such as TYR and PHE (Supplementary Information, Figure 2a), formed π-π interactions with hexagonal carbon rings, and the elongated, planar groups on ARG (Supplementary Information, Figure 2b), ASP and LYS aligned with the regular surface of the SWCNT. We report the energies of interaction and percent of interacting residues in Table 1.

The lowest energy SWCNT-actin complex (Figure 5a) showed SWCNT interactions with two actin monomers with an orientation of the SWCNT close to the actin groove. However, this interaction did not represent the lowest potential energy of the system since the presence of the SWCNT slightly alters the conformation of actin subunits within the filament. Therefore, the simulation with the lowest potential energy (Figure 5f) had a minimum alteration of actin subunits within the filament. Thus, there appears to be a dynamic instability between the optimum conformation of the actin filament and the optimum interaction between SWCNTs and the actin filament. Since the surface chemistry of the SWCNT is mostly uniform, the SWCNT is able to translocate in one dimension along the three monomer filament.

We suggest that a small, non-specific interaction of the SWCNT with the available face of the actin monomers may be insufficient for stable association. However, a sliding behavior of SWCNTs along the filament may allow a continuous association of SWCNTs along F-actin (see Shams *et al*. for details [49]). This interaction may be due, in part, to similar anisotropic properties (e.g., L_c_/D >> 1) of SWCNTs and F-actin (Table 2). Specifically, the persistence lengths of F-actin and SWCNTs are nearly similar, suggesting similar length scales of entropic fluctuations.

**Table 1:** The energies of interaction and percent of interacting residues from the molecular dynamics simulations of SWCNT/actin interaction.

	Charged					Polar					Hydrophobic					
Amino acid	Asp	Arg	Glu	Lys	His	Ser	Asn	Gln	Thr	Tyr	Ile	Gly	Pro	Ala	Phe	Val
Energy (−kJ/mol)	26.4	31.4	19.7	18.7	19.0	29.6	25.2	28.1	27.1	47.0	37.4	10.4	20.7	14.0	32.7	18.8
% interacting residues	19	13	8	6	4	6	2	2	2	2	12	8	8	4	4	2

**Table 2:** Selected mechanical properties of SWCNTs and F-actin.

	F-actin	SWCNTs
L_p_ (μm)	17	22
D (nm)	6	0.7–1.3
L_c_ (μm)	~(10)	0.150
E (GPa)	1	1,000

#### 2.1.4. SWCNT-Actin Interactions

Through the SWCNT-induced change in fluorescence lifetime, we have shown direct interactions (within 5 nm) of SWCNTs and purified actin filaments produced *ex vivo*, and intracellular FLIM results have suggested that SWCNTs and F-actin directly interact within cells. We suggest that the similarity in diameters and persistence lengths of F-actin and SWCNTs is responsible for the preferential interaction. Cells have a large concentration of F-actin, and we have observed delivery of up to 10^6^ SWCNTs per cell [45]. These high concentration constituents associate via weak protein-SWCNT interactions, and the global strength of the interaction, is additive along the SWCNT interface with the repetitive actin filament. The anisotropic SWCNT is able to travel in one dimension along F-actin and maintain regions of semi-stable interaction. This dynamic association can then stabilize relatively short actin filaments (Figure 1) within the cell away from basal stress fibers and in apical F-actin structures (Figure 3). Also, SWCNT-F-actin interactions stabilize inter-actin bundling via lateral interactions, leading to the observed cellular phenotypes. The SWCNT-F-actin interaction does not appear to impact F-actin-myosin II interactions; there are no localization changes of myosin II except in combination with highly distorted F-actin structures.

### 2.2. SWCNTs Alter F-Actin Function

To examine what downstream effects SWCNTs have on cells by altering actin, we considered actin-mediated cellular functions. Previously, we have shown altered cell division in cells treated with high levels of SWCNTs-PF127 [30]. Specifically, we observed arrested cytokinesis at the last stage of mitosis, wherein reorganization of the actin cytoskeleton is required for the formation and final disassembly of the cytokinesis cleavage furrow. 

#### 2.2.1. Myosin II Partially Redistributes with Altered F-Actin Structures

**Figure 6:**
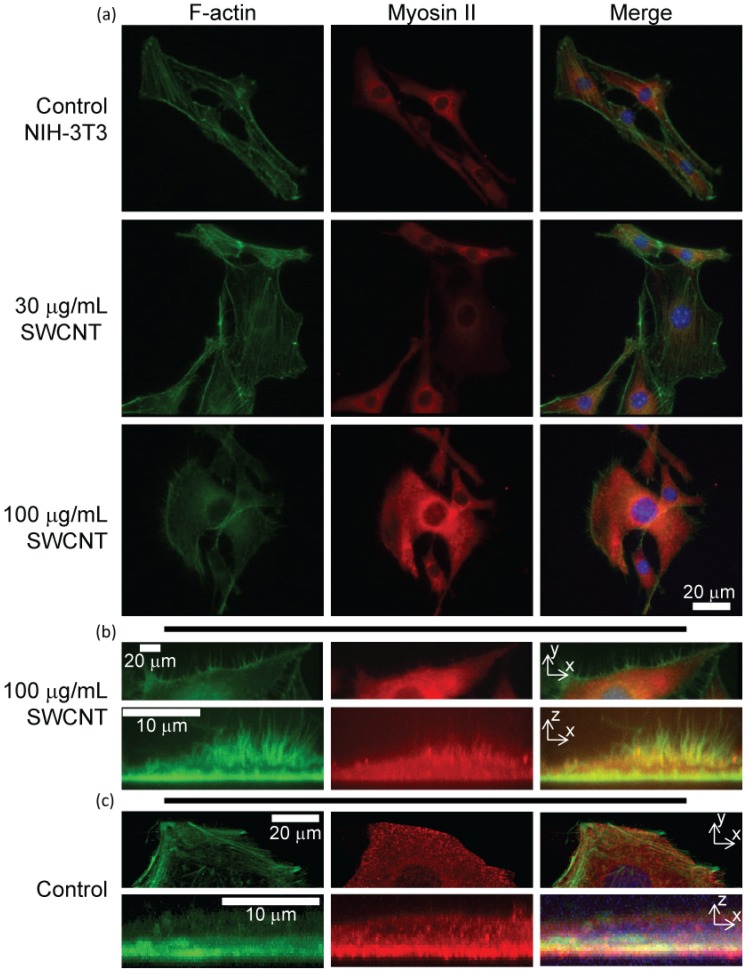
Imaging of myosin II in NIH-3T3 cells: (**a**) Widefield fluorescence images show that myosin II regulation is only slightly affected by incubation with SWCNTs-PF127, although F-actin is alerted; (**b**) Confocal images of a SWCNT-treated (100 μg/mL) NIH-3T3 cell compressed in Z (top) and Y (bottom) show F-actin outward protrusions and apical protrusions, respectively. These protrusions are mostly devoid of myosin II; (**c**) Control images of NIH-3T3 fibroblasts show more defined edges with actin and myosin primarily at the basal region of the cell.

Since actin filaments are altered and redistributed inside SWCNT-treated cells, we examined myosin II localization in SWCNT-treated cells. We observed a minority of myosin II mislocalized throughout the cell, but myosin II mislocalization appeared to occur colocalized to altered F-actin structures (Figure 6). Specifically, we examined myosin II and F-actin mislocalization within peri-cellular radial protrusions (Figure 6b). We measured F-actin and myosin II in peri-cellular projections from the central cell body, which are common in isolated cells treated with SWCNTs. Most F-actin projections did not have myosin II contained within them. In the minority of projections that contained myosin, the myosin extended only 36 +/− 6 % of the length of the F-actin. F-actin was able to extend into long projections (in some cases > 10 μm), extending apically from the surface and radially from the cell, but myosin II is not recruited to these regions. Together, these results suggest that F-actin is reorganized in the presence of SWCNTs, and a small amount of myosin II is reordered along with the F-actin.

#### 2.2.2. SWCNT Treatment Alters Cell Force Generation

**Figure 7:**
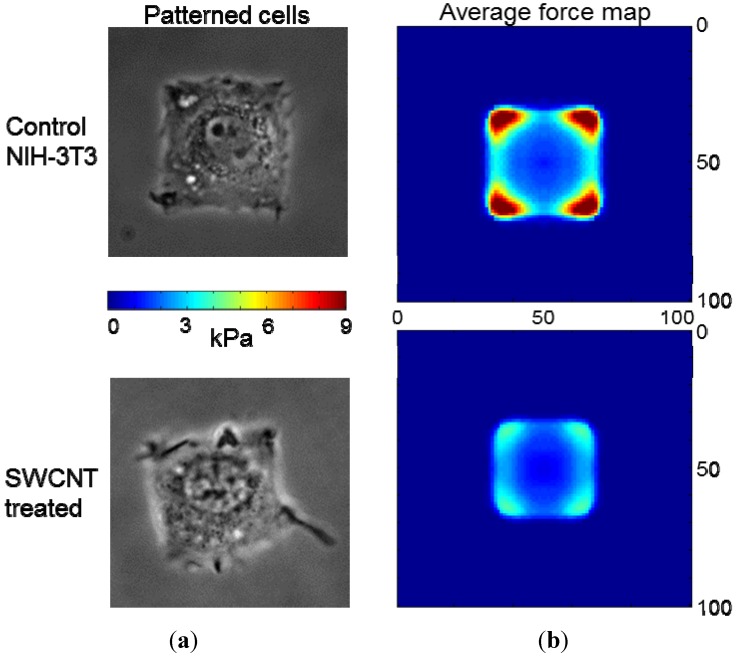
Fibroblast force generation; (**a**) Phase-contrast images of control and SWCNT-treated (200 μg/mL for 24 hr) NIH-3T3 cells confirmed that the cells were well-adhered and spread to fill the micropatterned gelatin square surface on the polyacrylamide hydrogel; (**b**) Heat map of traction force averaged over n = 16 for treated cells and n = 20 for control cells demonstrating that SWCNTs reduce the magnitude but do not alter the distribution of traction force.

With reorganized actin filaments and associated myosin II, we considered altered cellular force generation from redistributed actomyosin machinery. We patterned adhesive regions (40 μm × 40 μm squares; Figure 7) on deformable substrates with embedded tracers and allowed NIH-3T3 fibroblasts to attach and produce traction on the substrate. We were able to visualize the force generated by the cell by imaging deformation of the substrate before and after rapid cellular detachment. Our previous studies examined force generation capabilities with comparisons between individual cells or averaged mechanics [30]. However, the regular geometry of cells allowed for composite overlays of many SWCNTs-PF127-treated and control cells. We found that SWCNT treatment of fibroblasts reduces average traction force of the cells by half (Figure 7). We suggest that this reduction in traction force is due to loss of larger actin filaments which transect the cell allowing for larger forces.

#### 2.2.3. Cellular Functions Altered by High Levels of SWCNT-F-actin Interactions

In previous studies, we observed altered cellular phenotype, including shape changes, reduced proliferation and altered cytokinesis associated with high levels of SWCNTs-PF127 [30]. We also observed reduced force generation (Figure 7). However, we suggest that these changes are primarily associated with altered F-actin structures. Myosin II only appears mildly displaced in cells treated with high levels of SWCNTs-PF127. Myosin II is only displaced with actin filaments, and myosin II appears less altered than F-actin (Figure 6).

## 3. Experimental Section

### 3.1. Synthesis of SWCNTs

SWCNTs were purified, length fractionated and dispersed as previously described [30,50,51,52]. The final product was highly purified, length-selected (145 +/− 17 nm), pristine HiPCO SWCNTs, with an average diameter of 1 +/− 0.3 nm [30]. 

### 3.2. Cell Culture

#### 3.2.1. HeLa

HeLa cells were grown in Dulbecco’s Modified Eagle’s Medium (DMEM), low glucose (Hyclone catalog number SH30021) supplemented with 10% v./v. fetal bovine serum (Invitrogen) and 1% v./v. penicillin/streptomycin (Invitrogen) at 37 °C and 5% CO_2_. Upon culture reaching ~90% confluency, cells were subcultured and, for imaging experiments, seeded on sterilized #1.5 glass coverslips at 3.0 × 10^4^ cells/cm^2^. After 24 hr, cells were exposed to SWCNTs-PF127 diluted in HeLa cell culture media to the indicated concentration for the indicated exposure time.

#### 3.2.2. NIH-3T3

NIH-3T3 cells were grown in DMEM, high glucose (Hyclone catalog number SH30022) supplemented with 10% v./v. newborn calf serum (Invitrogen) and 1% v./v. penicillin/streptomycin (Invitrogen) at 37 °C and 5% CO_2_. Cultures were maintained until reaching ~75% confluency (well before cells would form striations) and were then subcultured. Like HeLa cells, for fluorescence imaging experiments, NIH-3T3s were seeded on sterilized #1.5 coverslips at 3.0 × 10^4^ cells/cm^2^ and exposed to SWCNTs after 24 hr.

#### 3.2.3. hMSC

Human mesenchymal stem cells (hMCSs) (Stem Cell Technologies) were grown in GIBCO MesenPRO RS Basal Medium and Growth Supplement with 2 mM of L-glutamine at 37 °C and 5% CO_2_. hMSCs were not investigated beyond passage 7, and their complete media was not used for longer than 15 days. When hMSCs were near confluency, they were seeded at 5,000–6,000 cells/cm^2^ onto sterilized #1.5 coverslips. After ~72 hr, the hMSCs were ~50% confluent, and fresh media was added.

#### 3.2.4. Incubation with SWCNTs

After providing cells sufficient time to adhere to the substrate and to begin proliferating (typically 24 hr for HeLa and NIH-3T3 and 72 hr for hMSC), concentrated stock solutions of SWCNTs-PF127 were diluted in fresh, temperature-equilibrated cell culture media to their indicated final concentration which was added to the cell cultures.

### 3.3. Microscopy

#### 3.3.1. Fixation and Labeling

To prepare samples for fixed-cell imaging, the SWCNT-laden medias were aspirated; the cells were washed with 1× cell culture PBS; and the cells were incubated with 3.7% formaldehyde for 15 min. For fluorescent imaging, the fixed cells were permeabilized with 0.2% v./v. Triton X-100 for 5min. Cells were washed with PBS and then exposed to 0.25 μg/mL of 4',6-diamidino-2-phenylindole, dihydrochloride (DAPI) (labeling DNA), 0.165 μM of rhodamine phalloidin (labeling F-actin), Oregon Green phalloidin (labeling F-actin) and/or 0.3 μM DNase I (labeling G-actin) for 20 min. For myosin II labeling, the samples were first blocked with 0.2% BSA in PBS for 1 hr. Then, the primary Myosin II antibody (Nonmuscle Myosin Heavy Chain II-A Polyclonal Antibody Purified, Covance) was diluted 1:500 in PBS/0.2% BSA and incubated for 1 hr. Subsequently, the samples were washed 3× with PBS/0.2% BSA solution and subjected to a secondary Alexa Fluor 555 goat anti-rabbit antibody for 1 hr. The solutions were then aspirated, the cells washed, the coverslips mounted onto glass slides (Fisher) using 20 μL of mounting media (Fluoromount-G, Southern Biotech) and the edges sealed.

#### 3.3.2. Widefield and Confocal Fluorescence Imaging

Widefield fluorescence microscopy was performed using an inverted Leica DMI 6000B. Images were acquired using a 1.4 NA, 63×, oil immersion objective and a Leica DFC350 FX charge-coupled device (CCD). Confocal microscopy was performed using a Leica SP5 laser scanning confocal microscope with a pulsed, tunable, Ti-Sapphire Coherent Chameleon Laser and three visible lasers: Argon, HeNe 543 and HeNe 633. Confocal scanning was performed with a pixel resolution of 1,024 × 1,024 at an acquisition rate of 400 Hz using a 1.4 NA, 100×, oil immersion objective. 3D image compressions to 2D were performed using Leica Application Suite Advanced Fluorescence (LAS AF) software.

#### 3.3.3. FLIM

Time-correlated single photon counting fluorescence lifetime imaging microscopy (FLIM) was performed on a Leica TCS SP5 laser scanning confocal microscope with 1.4 NA, 100×, oil immersion objective using a pulsed, tunable, Ti-Sapphire Coherent Chameleon Laser and a Becker & Hickl SPC-830 photon counting device, as described previously [29]. Briefly, 256 × 256 pixel lifetime images were acquired for 180 s to enable accurate determination of two exponential decays and to minimize the coefficient of variation [48,53,54,55]. Fluorescence lifetime images were generated in SPCImage (Becker & Hickl), using binning, if necessary, to achieve peak photon counts of ≥1,000. The calculated single (τ_1_) and double exponential (τ_1_ and τ_2_) decay lifetimes, along with their relative magnitudes and corresponding goodness of fits, were analyzed in MATLAB [29]. For double exponential decays, we report the mean fluorescence lifetime (τ_m_), which is the weighted average of τ_1 _and τ_2_. All reported values of lifetime are the average value of lifetime averaged across all analyzed images per experimental condition.

#### 3.3.4. Traction Force Microscopy

Traction force microscopy was performed as described previously [36]. Briefly, fluorescent beads were embedded in ECM patterned polyacrylamide hydrogels of known stiffness to serve as fiducial markers of gel displacement. SWCNT-PF127-treated and control cells were seeded on top of the gels and allowed to spread overnight. Images of the beads were taken before and after the manual removal of the cell with a microneedle. Custom software was used to calculate bead displacement fields, which were then used to calculate traction stress images with the software package LIBTRC, courtesy of Micah Dembo (Boston University, Boston, MA, USA; [56]).

### 3.4. Filament Image Processing

The actin filament algorithm included a sequential execution of four estimation-inference steps. In the first step, we estimate the likelihood of pixels belonging to actin filaments and their rough orientation by filtering the original image with a bank of artificial filaments that differ in rotation, scale and curvature. We use the best response from the filters in conjunction with the original intensity to construct a filament likelihood (or enhanced) image. In the second step, we apply a foreground estimation algorithm similar to [57] to the filament enhanced image and obtain a binary image split into filament and non-filament portions. In the third step, we apply a morphological image thinning procedure, such as [58], to estimate filament centerline locations in the binary image. The final step disambiguates the filament bifurcation and intersections and measures the lengths of the individual filaments in the entire image. Numerical validation of actin length distribution was carried out on an image bank consisting of artificially generated filament networks with Gaussian distributed lengths (having pre-defined mean and standard deviation) and increasing filament density. Across all experiments carried out, the maximum relative error (error in filament count divided by the real count) for filament count in each image was within 3% and the maximum root mean square error in filament length was within 5 pixels (with an image size of 128 by 128 pixels, mean filament length being 50 pixels and standard deviation being 20 pixels). A more complete description of the method appears in [59].

### 3.5. Molecular Dynamics Simulation

The structure of actin was obtained from the protein data bank (PDB ID: 3LUE). A 1.1 nm long, armchair, (5,5)-SWCNT was generated and used in all simulations [60]. Molecular dynamics (MD) simulations were performed using the GROMACS software package and GROMOS96 force field [61,62]. The SWCNT-actin complex system was solvated in a triclinic box with TIP3P explicit water model. Periodic boundary conditions were imposed in all three directions. The extra charge of the system was neutralized by adding counter ions. The system was minimized using the steepest descent algorithm, and was equilibrated for 500 ps. The time step of 2 fs was used. The electrostatic and van der Waals interactions were modeled using the Particle Mesh Ewald method and SWITCH algorithm, respectively. The temperature was controlled with a Nose-Hoover thermostat at 300 K and the pressure was kept at 1 bar using a Berendsen barostat. The total time of each simulation was 5 ns. The Visual Molecular Dynamics (VMD) software was used for visualizations [63].

## 4. Conclusions

From the molecular to sub-cellular to cellular to functional levels, we have shown that SWCNTs preferentially interact with and alter F-actin. This work demonstrates some of the unique, important SWCNT sub-cellular interactions important to SWCNT biotechnology safety and development. Further, it suggests that SWCNTs can be used as a new type of actin altering agent, allowing for new fundamental biological and biophysical studies.

## Supplementary Files

Supplementary File 1 PDF-Document (PDF, 263 KB)
