# Supplementary material for: Altered Cell Mechanics from the Inside: Dispersed Single Wall Carbon Nanotubes Integrate with and Restructure Actin
Source: J Funct Biomater. 2012 May 23;3(2):398–417. doi: 10.3390/jfb3020398 (PMC4047933; doi:10.3390/jfb3020398)

*J. Funct. Biomater.* **2012**, *3*, 1-3; doi:10.3390/jfb3020398

OPEN ACCESS
*Journal of*
**Functional Biomaterials**
**ISSN 2079-4983**
www.mdpi.com/journal/jfb/

*Article*

# Altered Cell Mechanics from the Inside: Dispersed Single Wall Carbon Nanotubes Integrate with and Restructure Actin

**Brian D. Holt [1], Hengameh Shams [2], Travis A. Horst [1,3], Saurav Basu [1,4], Andrew D. Rape [1], Yu-Li Wang [1], Gustavo K. Rohde [1,4], Mohammad R. K. Mofrad [2], Mohammad F. Islam [5,*] and Kris Noel Dahl [1,3,*]**

[1] Department of Biomedical Engineering, Carnegie Mellon University, Pittsburgh, PA 15213, USA; E-Mails: bholt@andrew.cmu.edu (B.D.H.); thorst@andrew.cmu.edu (T.A.H.); sauravb@cmu.edu (S.B.); arape@andrew.cmu.edu (A.D.R.); yuliwang@andrew.cmu.edu (Y.L.W.); gustavor@cmu.edu (G.K.R.)

[2] Department of Bioengineering, University of California, Berkeley, CA 94720, USA; E-Mails: hengameh@Berkeley.edu (H.S.); mofrad@berkeley.edu (M.R.K.M.)

[3] Department of Chemical Engineering, Carnegie Mellon University, Pittsburgh, PA 15213, USA

[4] Center for Bioimage Informatics, Carnegie Mellon University, Pittsburgh, PA 15213, USA

[5] Department of Materials Science and Engineering, Carnegie Mellon University, Pittsburgh, PA 15213, USA

* Authors to whom correspondence should be addressed; E-Mails: krisdahl@cmu.edu (K.N.D.); mohammad@cmu.edu (M.F.I.); Tel.: +1-412-268-9609 (K.N.D.); +1-412-268-8999 (M.F.I.); Fax: +1-412-268-7139 (K.N.D.); +1-412-268-7596 (M.F.I.).

*Received: 23 March 2012; in revised form: 1 May 2012 / Accepted: 15 May 2012 / Published: 23 May 2012*

---

**Figure S1.** (**a**) Modeling representation of a three actin monomers combined into a filamentous arrangement (*i.e.*, actin trimer) as a model segment of F-actin; (**b**) The initial configurations of the SWCNTs from the six simulations shown in Figure 5 (SWCNT color code is the same as Figure 5) with an insight to the position of the actin trimer with respect to F-actin.

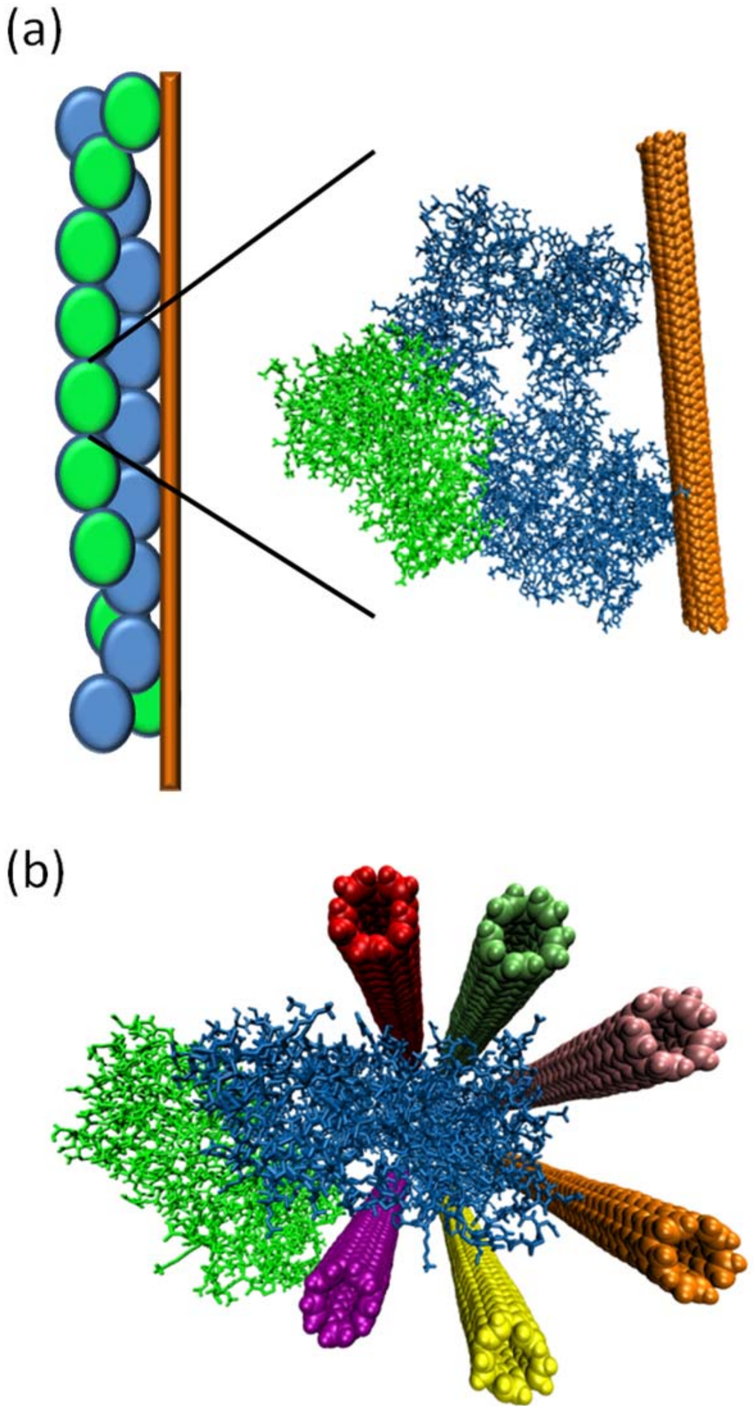

**Figure S2.** Two important representative surface interactions of actin residues (**a**) PHE and (**b**) ARG with a SWCNT.

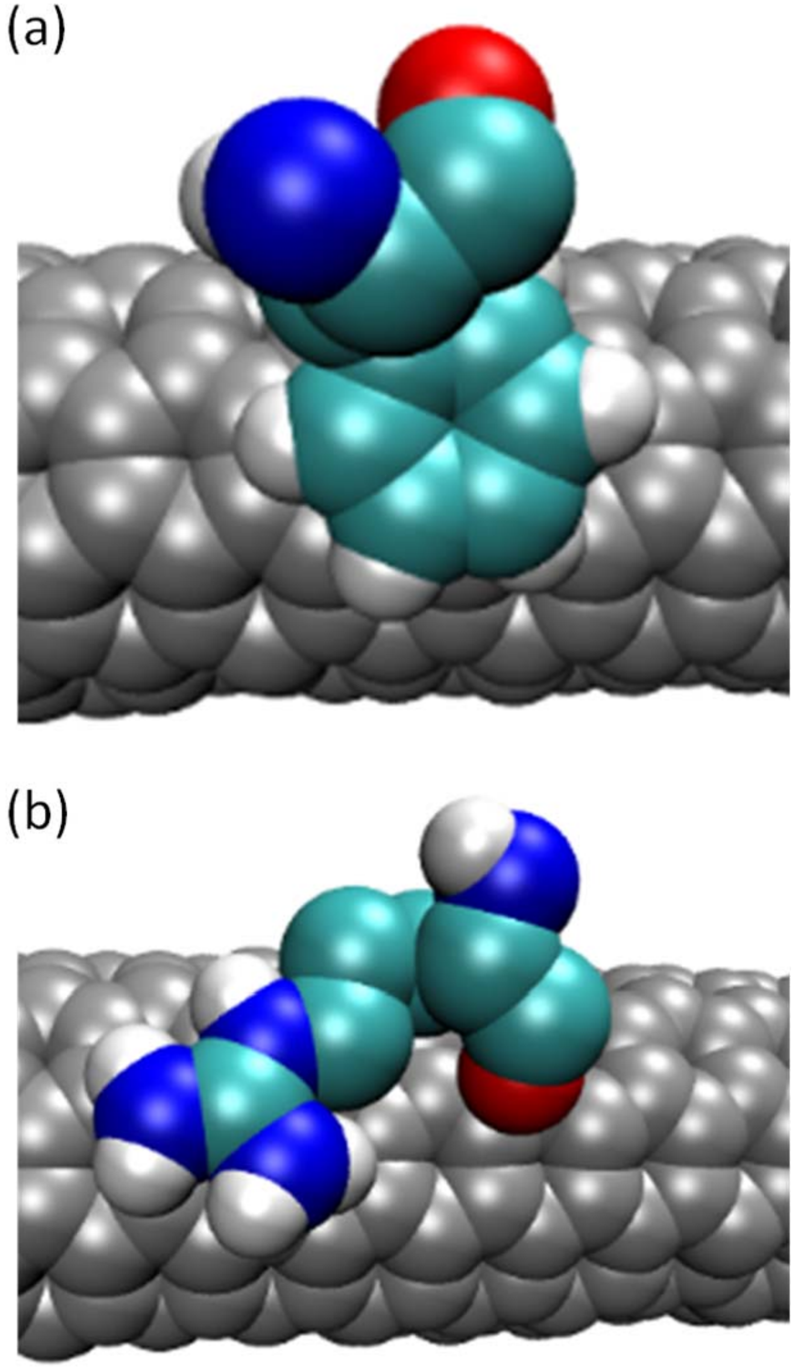

Supplement: Supplementary File 1 — PDF-Document (PDF, 263 KB) [file jfb-03-00398-s001.pdf]
